# Response Variance in Functional Maps: Neural Darwinism Revisited

**DOI:** 10.1371/journal.pone.0068705

**Published:** 2013-07-11

**Authors:** Hirokazu Takahashi, Ryo Yokota, Ryohei Kanzaki

**Affiliations:** 1 Graduate School of Information Science and Technology, The University of Tokyo, Bunkyo-ku, Tokyo, Japan; 2 Research Center for Advanced Science and Technology, The University of Tokyo, Meguro-ku, Tokyo, Japan; 3 PRESTO, JST, Kawaguchi, Saitama, Japan; University of Salamanca- Institute for Neuroscience of Castille and Leon and Medical School, Spain

## Abstract

The mechanisms by which functional maps and map plasticity contribute to cortical computation remain controversial. Recent studies have revisited the theory of neural Darwinism to interpret the learning-induced map plasticity and neuronal heterogeneity observed in the cortex. Here, we hypothesize that the Darwinian principle provides a substrate to explain the relationship between neuron heterogeneity and cortical functional maps. We demonstrate in the rat auditory cortex that the degree of response variance is closely correlated with the size of its representational area. Further, we show that the response variance within a given population is altered through training. These results suggest that larger representational areas may help to accommodate heterogeneous populations of neurons. Thus, functional maps and map plasticity are likely to play essential roles in Darwinian computation, serving as effective, but not absolutely necessary, structures to generate diverse response properties within a neural population.

## Introduction

Functional maps are commonly found in the sensori-motor cortices in the forms of columnar organizations [1]–[3]. These maps are subject to change during associative or skill learning [4]–[7]. Despite a broad consensus on the existence of plasticity in cortical organization, the mechanisms by which functional maps and map plasticity contribute to cortical computation remain controversial. Learning-induced map plasticity has been interpreted as evidence that cortical representations encode behaviorally relevant information [7]–[11]. However, several pieces of evidence argue against the functional significance of columnar organization. First, the capricious expression of ocular dominance columns suggests that the cortical column is a structure without any function [12], [13]. Second, map plasticity is not always associated with enhanced perceptual ability [14]–[16], and is sometimes associated with deteriorated percepts [17]. Lastly, neural plasticity is transient in motor and perceptual learning, thereby contradicting the notion that map plasticity is crucial to learning [18]–[20]. Thus, it remains unclear how learning-induced plasticity is related to cortical map structure.

More recent experiments have demonstrated that map expansion in the auditory cortex improves perceptual learning but is not necessary for improved performance [21]. Furthermore, we previously found that the learning-induced map plasticity in the auditory cortex is dependent on the learning stage. In appetitive operant conditioning tasks, the tone-responsive area globally expands during the early stage of learning, but shrinks during the late stage [22], [23]. These findings are reminiscent of neural Darwinism, which predicts that variation and selection within neural populations are crucial to cortical computation [4], [24], [25].

The Darwinian principle may also be supported by recent reports of heterogeneity among similar computational units. Two-photon calcium imaging in the auditory cortex has revealed significant heterogeneity in stimulus encoding among neurons in close proximity within a single column. Thus, while a tonotopic map is evident macroscopically, the map exhibits significant variability within a single column [26], [27]. Such heterogeneity within a column is also consistent with our recent findings that the degree of variation of stimulus encoding among multi-unit responses is dependent on tonotopic columns [28]. Furthermore, learning enhances sparse network coding, which makes the response properties of individual neurons more distinct from one another [29]. Thus, a growing body of evidence suggests that neural Darwinism plays a role in the sensori-motor cortex.

However, experimental proof of whether and how the cortical map is related to heterogeneity of neurons in Darwinian computation has not yet been reported. Here, we hypothesize that response variance within a given computational unit (e.g., a tonotopic column and functionally delineated field) is closely associated with the size of the corresponding unit. We tested this hypothesis by using learning-stage-dependent, field-specific map plasticity data gathered in the auditory cortex of rats. We considered two possible outcomes of our data analysis. First, overrepresentation in the cortical map may be a consequence of accommodating a heterogeneous population of neurons within a given functional unit. In this case, response variance among neurons may increase with the size of the representational area. Alternatively, overrepresentation may be a sign of redundant encoding, in which important information is represented by more neurons than necessary. In this case, response variance may not change with learning-induced map plasticity. Our analysis suggests that the former is the case in the auditory cortex of rats.

## Results

The data shown here were partially reported in our previous study in which we used tones to evaluate map plasticity in appetitive operant training in the auditory cortices of anesthetized rats [23]. In the training, rats were rewarded for nose-poking during the presentation of a conditioned stimulus (CS) of 20-kHz tone. The rates of both hit and false-positive responses increased during the early stage of training, while hit responses continued to increase but false positive responses decreased thereafter. On day 4, the maximum false-positive rate was observed. Both hit and false positive rates reached asymptotes by day 20. At the recordings, 8 rats had engaged in the training for 4 consecutive days (Day 4 group), and 8 other rats underwent training for 20 days or more (Day 20 group). Another 8 rats were assigned to a non-trained, naïve control group. The total numbers of tone responsive sites were 838, 965, and 618 in the naïve, Day 4 and Day 20 groups, respectively.

**Figure 1:**
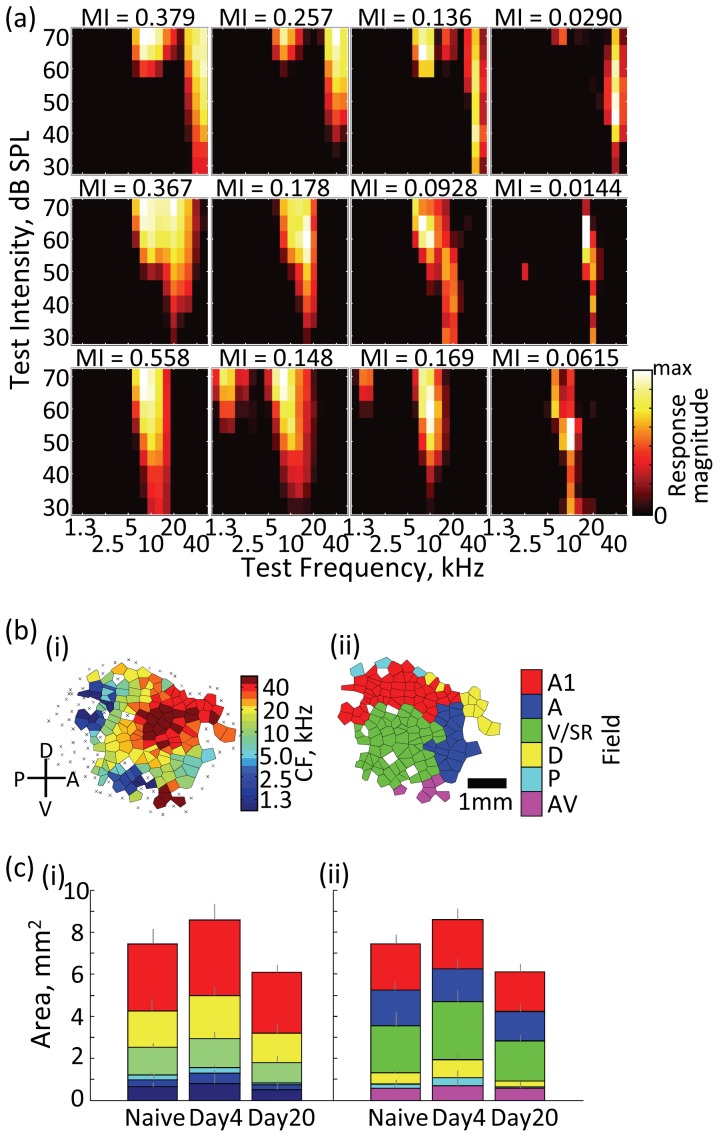
Characterization of neural responses in the auditory cortex. (a) Representative frequency response area (FRA). Mutual information (MI) for each unit is indicated in the upper of insets. (b) Representative functional map in the rat auditory cortex: (i) tonotopic map; and (ii) auditory field map. The primary, anterior, ventral/suprarhinal, posterior, dorsal, and antero-ventral auditory fields are labeled as A1, A, V/SR, P, D, and AV, respectively. (c) Learning-stage-dependent representational area. Functional maps were quantified in the naïve group (n = 8) and conditioned groups at the early (day 4; n = 8) and late stages (day 20; n = 8). The conditioning groups were rewarded by nose-poking at the presentation of 20-kHz tone.

Conventionally, frequency response areas (FRAs) are characterized to identify the characteristic frequency (CF) at each recording site and the tonotopic map in the auditory cortex (Fig. 1a). The tonotopic map is then further divided into multiple auditory fields on the basis of tonotopic discontinuity, response latency, and FRA properties (Fig. 1b). These tonotopic maps and field maps changed dynamically during learning (Fig. 1c).

**Figure 2:**
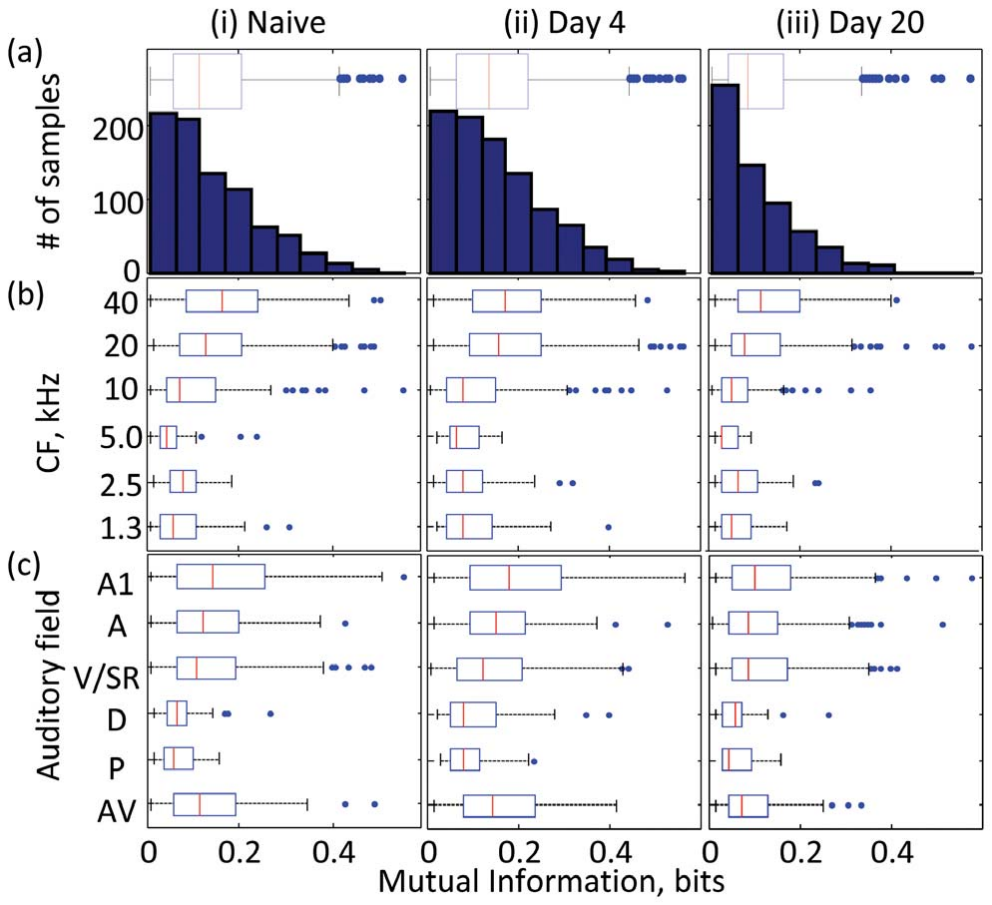
Distribution of mutual information. (a) Histogram of entire data: (i) naïve (n = 838); (ii) day 4 (n = 965); and (iii) day 20 (n = 618). (b) CF-dependent distribution. On each box, the central mark is the median, and the edges of the box are the 25th and 75th percentiles. The whiskers extend to the most extreme data points (maximum and minimum values) not considered outliers, which are larger than the 75th percentiles or smaller than the 25th percentiles by 1.5 times the inter-quartile range. (c) Auditory-field-dependent distribution.

Such conventional characterization has largely ignored the role of response variability. Despite having an identical CF, some multiunit responses show large FRAs (left column in Fig. 1a), while others show small and uncertain FRAs (right column in Fig. 1a). To quantify such variability among units, we used mutual information (MI) to estimate how a discharge rate at a given site carried information about the frequency of test tones. Qualitatively, small and uncertain FRAs tended to result in small MI. Consistent with the notion of sparse coding in the auditory cortex, MI was typically small. However, the degree of response variance was dependent on CF and auditory field (Levene test, p<1.0e-12), with more units having large MI in high CF regions and A1 (Fig. 2; Kruskal-Wallis test, p<1.0e-8). Additionally, MI distribution also depended on the stage of learning (two-sample Kolmogorov-Smirnov test with Bonferroni correction, p<0.01; Kruskal-Wallis test, p<1.0e-15; Levene test, p<0.01); multiunit responses with high MI emerged at day 4, and thus response variation increased, whereas these high-MI units decreased at day 20. These results were consistent with the variation and selection process in neural Darwinism. Consequently, neural representations may become sparser during the later stages of learning [29].

**Figure 3:**
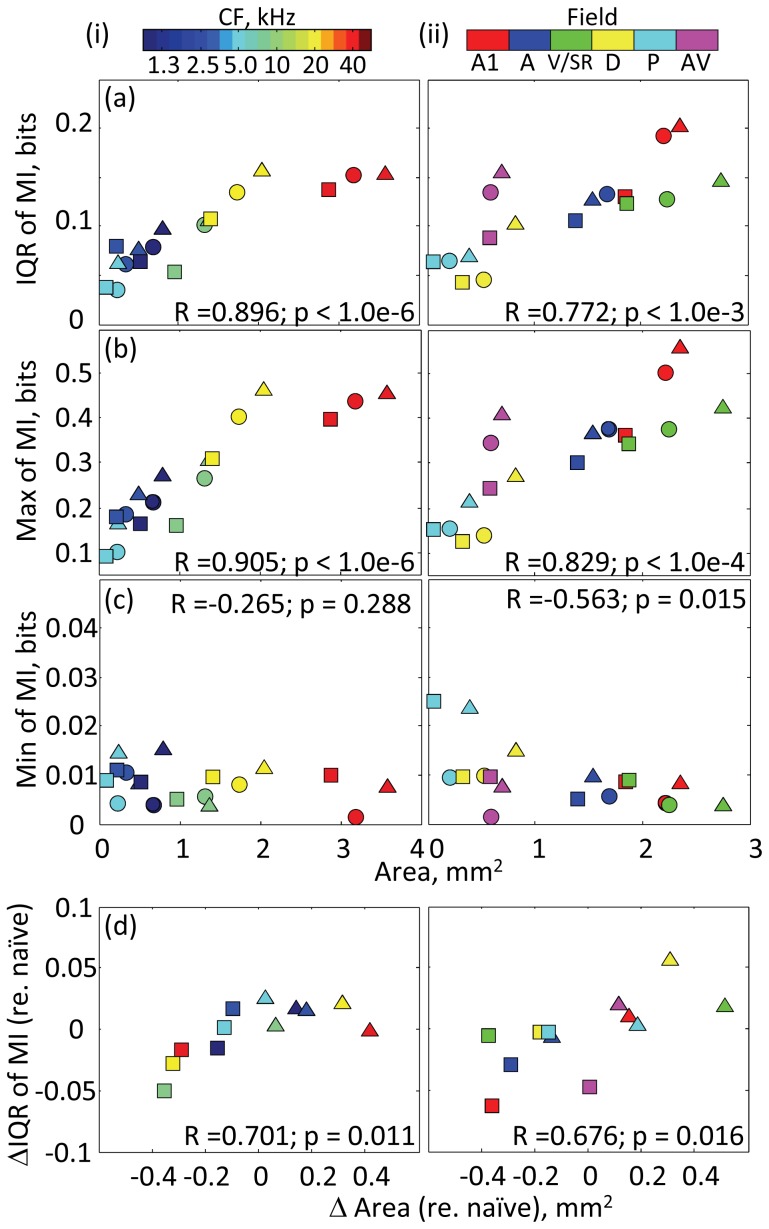
MI distribution on the functional map. (a) Interquartile range (IQR) of MI with respect to representational area with a given property: (i) CF; and (ii) Auditory field. The shapes of symbols indicate the test groups: circle, naïve; triangle, day 4; and square, day 20. R and p are Pearson’s correlation and its significance level (t-test). (b) Maximum value of MI. (c) Minimum value of MI. (d) Learning induced changes of MI IQR (ΔIQR) and representational area (Δarea).

**Figure 4:**
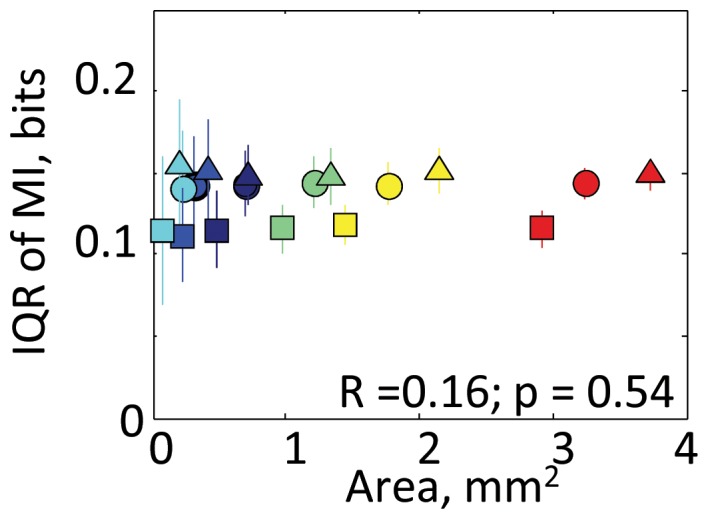
Validation that the positive correlation between the representational area and MI variance is caused by frequency-dependent MI distribution, but not by inadequate sampling. IQR was estimated as a function of the representational area in shuffled data, which were randomly resampled irrespective of any properties. The shapes of symbols indicate the test training groups: circle, naïve; triangle, day 4; and square, day 20. Representational areas of each resampled dataset were determined on the basis of the tonotopic map in each training group. Means and standard deviations are shown when the resampling was conducted 100 times. No significant correlation between IQR and representational area was observed, thereby denying the possibility that IQR may increase with the number of samples because test datasets are inadequately small.

This MI distribution is likely linked to properties of the functional map. Irrespective of the learning stage, the variance of MI was significantly positively correlated with the representational area delineated by either CF or auditory fields (Fig. 3a; t-test, p<0.001). This positive correlation between the representational area and MI variance was not an artifact due to inadequate sampling (i.e., inadequately small dataset), but is instead caused by frequency-dependent MI distribution (Fig. 4); there was no correlation between the representational area and MI variance in shuffled data, which were randomly resampled irrespective of any properties (p>0.5). This analysis has excluded the possibility that IQR increased with the number of samples. Significant correlation was also observed in the maximum value of MI (Fig. 3b; p<1.0e-4), but was not obvious in the minimum value (Fig. 3c) because small MI was commonly observed in all test groups. Furthermore, the learning-induced changes of representational area were also positively correlated with those of MI (Fig. 3d; p<0.05), suggesting that a gain and loss of representational area are associated with diversification and sparsification of neural responses, respectively. This result adds compelling evidence regarding the functional link between the response variability and representational area because the measure of learning-induced change is not biased by the delineation of the test area, i.e., binning of CF and field.

**Figure 5:**
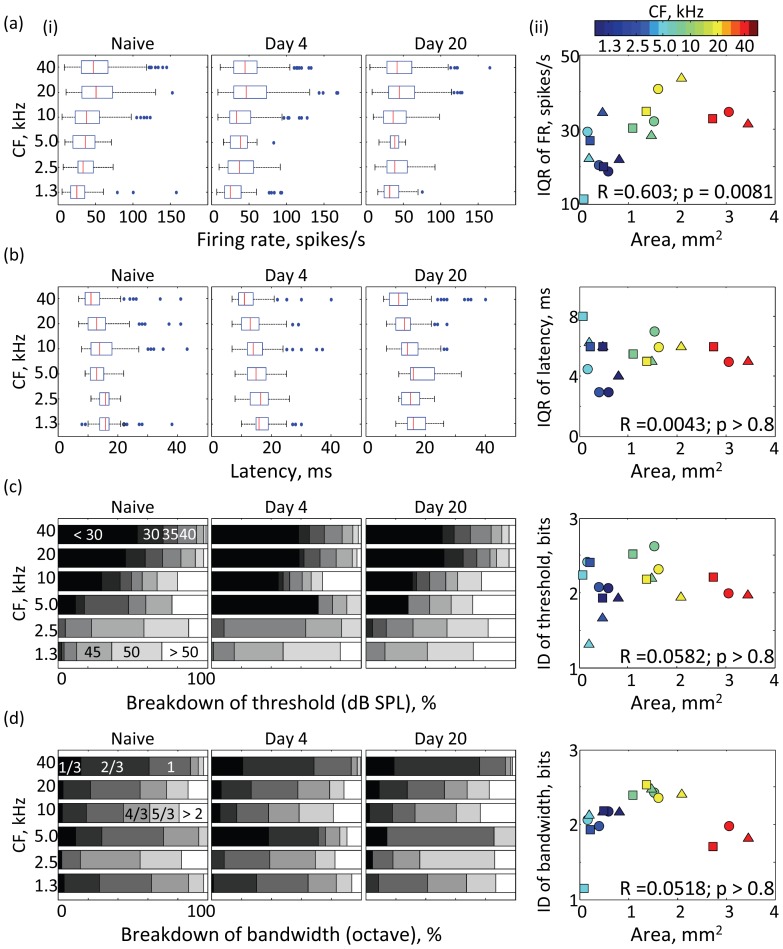
Distribution of conventional properties in tone-evoked neural responses and FRA. (a) Peak firing rate; (b) Peak latency; (c) Threshold; (d) Bandwidth. (i) CF-dependent distribution in each test group (naïve, day4 and day 20). Breakdown lists, instead of boxplots, are used to characterize the threshold (c) and bandwidth (d) because these properties are discretized. Proportions of subgroups of threshold (in dB SPL) and bandwidth (in octave) are shown in discrete grey levels as indicated in the left inset. (ii) Variance of response property with respect to representational area with a given CF. IQR was used to quantify the variances of firing rate (a) and latency (b), whereas the Shannon index of diversity (ID) was used in discrete properties of the threshold (c) and bandwidth (d). Other conventions comply with Fig. 3.

Lastly, for comparison to the MI distribution discussed so far, Fig. 5 characterizes CF-dependent variance of conventional properties of neural responses and FRA, i.e., peak firing rate, peak latency, threshold and bandwidth. All of these parameters showed CF-dependent distributions. For example, the variance and median of peak firing rate was dependent on CF (Fig. 5a (i); Levene test, p<0.001; Kruskal-Wallis test, p<0.01). The peak latency was shorter in high-CF regions than in low-CF regions (Fig. 5b (i); Kruskal-Wallis test, p<1.0e-17). The threshold was lower in high-CF regions (40 kHz) than in low-CF regions (1.3 kHz) (Fig. 5c (i); two-sample Kolmogorov-Smirnov test, p<1.0e-17). The bandwidth was narrower in high-CF regions (40 kHz) than in low-CF regions (1.3 kHz) (Fig. 5d (i); two-sample Kolmogorov-Smirnov test, p<1.0e-5). Additionally, similar to MI distribution, the variance of peak firing rate was significantly positively correlated with the representational area delineated by CF (Fig. 5 a (ii); t-test, p<0.01). However, other parameters did not significantly correlate with the representational area (Fig. 5 b–d (ii); p>0.8), indicating that the links between the functional map and response variance is only found in specific response properties.

## Discussion

We have demonstrated that functional maps and plasticity in the auditory cortex are closely correlated with the variance of MI and firing rate of neural activity. Within the functional units of computation (e.g., tonotopic columns and auditory fields), the degree of response variability is likely to be co-modulated with the representational area according to training and experience. In other words, larger representational areas may help to accommodate a heterogeneous population of neurons that emit diverse responses to stimuli. These results suggest that the functional map plays an important role in implementing Darwinian principles in cortical computations. Our model is able to account for functional roles as well as some specific features of cortical map and map plasticity.

Moreover, except for the peak firing rate, the traditional response properties such as peak latency, threshold and bandwidth were not co-modulated with the representational area. This result was consistent with that of our previous study showing that MI and these response properties did not show clear correlation [28]. These traditional properties also varied among neurons, but we had problems interpreting how such variability contributes to the cortical computation of encoding tones. MI is more interpretable in terms of encoding because MI is rigorously defined as the reduction in uncertainty about the stimulus after a single neural response is observed [30]. Thus, our goal in this study was to quantify the variability in encoding ability of tone frequency among neurons and to investigate the link to the size of either tonotopic column with a given CF or functional auditory fields. Furthermore, it is worth noting that the variability of MI is caused by the amount of information conveyed by neurons (i.e., total entropy) rather than by the transmission efficiency [28]. Therefore, our model may be generally applicable to encoding of other stimulus properties beyond tone frequency.

Previous studies have emphasized on the variability of neural responses among single neurons [26], [27]. Considering that multiunit activities served as a spatially averaging filter of single-unit activities, the variance of single units should be properly reflected in the variance of multiunit activities. Thus, we believe that our results are associated with the heterogeneity of neurons within a given representational area. Alternatively, the fact that our hypothesis has been confirmed in multiunit data suggests that microcircuits within a cortical column rather than single units are the relevant computational unit. Further investigation is needed to address these alternative possibilities. Additionally, our findings should be tested with a more complex stimulus set, including natural sounds and vocalizations, because the simple stimulus set of tones used in this study has limited the evaluation of response heterogeneity, and thus, the actual effect might be larger than that reported here [31], [32].

Empirically, representational areas in the sensori-motor cortices are likely to increase with their functional importance [1], [7], [11]. For example, in the auditory cortex of rats, the ultrasonic, high-frequency region is wider than the low-frequency region (Fig. 1c), possibly because ultrasonic sounds are used for vocal communications [33], [34]. Similarly, a larger representational area is devoted to low frequencies below 10 kHz in the auditory cortex of cats [35] and monkeys [36], whose vocal communications are mediated within these lower ranges that are audible to humans. The biased distributions of functional columns can be taken as experimental evidence that functional maps represent an adaptive infrastructure in Darwinian computation, in which heterogeneous neural responses make computation more efficient.

The response variability, and thus the tone responsive area, tentatively increased in the early stage of learning and then decreased in the late stage (Fig. 2c). This is generally consistent with the expansion then renormalization model based on Darwinian selection [4], [24], [25]. The original theory of neural Darwinism involves selective death of neurons [24]; however, this is unlikely in our experiments because the size of the tone-responsive area not only shrank but also expanded during the learning. Instead, we should assume selective strengthening or weakening of population of synapses [4], [25]. In this model, the number of neural circuits that respond to task stimuli increases at the early stage, causing heterogeneous circuits to emerge. The late stage of learning constitutes selection of the most efficient neural circuit from the heterogeneous population. By the end of learning, the useful circuit is stabilized and the cortical map is normalized. This model is also consistent with recent findings in human studies that neural plasticity is transient in motor and perceptual learning [18]–[20].

We demonstrated that in the early stage of learning, the increase of response variability is associated with the emergence of large MI responses, which showed large, robust FRAs. These neural populations indicate increased responses to task-relevant stimuli. The most likely mechanism of this transient plasticity is disinhibition, which unmasks weak excitatory inputs, triggers Hebbian plasticity, and facilitates global remodeling of cortical map [37]. Such disinhibition is enabled by the activation of the cholinergic nucleus basalis, which also induces map expansion that improves learning [4], [21], [38]–[40].

By the end of learning, the cortical network was more dominated by small MI responses than the naïve cortex, suggesting that the effect of extended learning goes beyond renormalization: the learning makes cortical representation sparser and thus endows the cortical circuits with energy-efficient encoding [41]–[43]. This is also supported by a recent 2-photon imaging study demonstrating that associative learning enhances sparse population coding, by which the total network activity decreases [29]. Such modification is likely mediated by inhibitory synapses, which suppress responses to task-irrelevant stimuli [38], [44], [45].

The functional map endows neurons in close proximity with shared synaptic inputs, but allows for different outputs through mutually independent computations [26]. Decorrelating neuronal activities from shared inputs is a possible neural underpinning of such computation [46]–[48]. Thus, the functional map is likely to play an essential role in Darwinian computation, serving as an effective, but not absolutely necessary, structure to generate various response properties within a neural population.

## Materials and Methods

The original experimental data has already been published and described in detail in our previous work [23]. The estimation of MI and the validity of analyses were also described in detail elsewhere [28].

The animal experiments were carried out in strict accordance with the “Guiding Principles for the Care and Use of Animals in the Field of Physiological Science” by the Japanese Physiological Society. The protocol was approved by the Committee on the Ethics of Animal Experiments at Research Center for Advanced Science and Technology, The University of Tokyo (Permit Number: RAC07110). All surgeries were performed under isoflurane anesthesia, and all efforts were made to minimize suffering.

### Subjects

Twenty-four male Wistar rats at postnatal week 12–15 were used in total. At the time of recordings, 8 rats had engaged in behavioral training for 4 consecutive days (Day 4 group, or early-learning-stage group), and 8 other rats had trained for 20 days or more (Day 20 group, or late-learning-stage group). The remaining 8 rats were assigned to a naïve control group.

### Training

On the first day of training, body weights of rats were maintained at 85% of the normal weight through diet restriction for 5–7 days. Rats were rewarded for nose-poking during the presentation of a conditioned stimulus (CS). This training was performed in a custom-made operant chamber (O’hara & Co. Ltd., Tokyo, Japan) with a dimension of 20×24×35 cm. The chamber was equipped with a 3-cm-diameter poking hole and a food dispenser on the wall. A speaker on the ceiling of operant chamber delivered CS tone with a frequency of 20 kHz, intensity of 80 dB SPL (sound pressure level in decibel with respect to 20 µPa; <70 dB SPL in the operant chamber), and a duration of 3 s. The interval of CS presentation was determined pseudo-randomly within a range of 15–25 s. In a daily training session, the CS was presented 60 times in total. Nose-poking responses during the CS tone were scored as a hit, while responses in the absence of the CS were scored as a false positive. A hit triggered delivery of a 20-mg sucrose pellet.

At the early stage of learning, both the hit and false-positive rate increased. The false positive rate was maximum at day 4 and decreased thereafter. On day 20, both hit and false-positive rates were stable.

### Electrophysiological Mapping

Rats were anesthetized with isoflurane (3% at induction and 1–2% for maintenance) and were fixed using a custom-made head-holding device. Atropine sulfate (0.1 mg/kg) was administered at the beginning of the surgery and every 8 h thereafter. The temporal muscle, cranium, and dura overlying the auditory cortex were surgically removed and the exposed cortical surface was covered with silicone oil to prevent desiccation.

Acoustic stimuli were composed of tone bursts with 5-ms plateau and 5-ms rise/fall times. The test frequencies ranged between 1–50 kHz with 1/3-octave increments (1.0, 1.3, 1.6, 2.0, 2.5, 3.2, 4.0, 5.0, 6.4, 8.0, 10, 13, 16, 20, 25, 32, 40 and 50 kHz) and intensities between 30 and 70 dB SPL with 5-dB increments. Each tone was presented 20 times in a pseudorandom order. These stimuli were delivered at a left (contralateral) pinna every 200 ms through a sound delivery tube of an electrostatic speaker (Tucker-Davis Technologies, Inc., EC1). Prior to the experiments, acoustic calibration of each test tone was performed with a 1/4-inch microphone (Brüel and Kjaer, 4939).

Multiunit activities were recorded with teflon-coated tungsten microelectrodes (California fine wire Co.) at a depth of 500–800 µm from the pial surface. Each insulated probe had a diameter of 50 µm in total with a bare metal diameter of 30 µm (∼100 kΩ impedance at 1 kHz). The neural signals were obtained with an amplification gain of 1000, digital filter bandpass of 0.75–7.5 kHz and sampling frequency of 30 kHz (Cyberkinetics Inc.; Cerebus Data Acquisition System).

### Data Analysis

All analyses were performed offline with custom-written Matlab programs (The Mathworks, Natick, MA).

From multiunit activities, the peak latency of tone-evoked responses was determined as a temporal property at each recording site on the basis of a post-stimulus time histogram (PSTH) with a bin width of 1 ms. PSTH plotted a firing rate as the mean number of spikes per second evoked for the entire stimulus conditions. The peak latency was defined as the time bin in which the maximum number of spikes was recorded. The peak firing rate in PSTH was also determined to characterize each recording site.

The frequency response area (FRA) at each recording site was then determined on the basis of multiunit activities within 40-ms post-stimulus latency in response to the 18 test frequencies at 9 sound intensities. The evoked response to each tone was quantified by subtracting the sum of the mean spontaneous rate and 20% of the peak firing rate from the mean firing rate. The mean spontaneous rate was defined as the firing rate during the first 3 ms after stimulus onset, averaged over all stimuli. As in our previous works [23], [28], the characteristic frequency (CF) was determined at each recording site as the test frequency that evoked a reliable response at the lowest intensity (i.e., threshold) or the largest response at 30 dB SPL, the minimum intensity used in this experiment. The bandwidth at 10 dB above threshold was also characterized as an octave distance between the upper and lower frequencies of the tuning curve; when the threshold was lower than 30 dB SPL, the bandwidth at 30 dB SPL was taken.

The borders of auditory fields were determined by the discontinuity of tonotopic and latency gradients [23], [49], [50]. A1 was first defined on the basis of short peak latency in the most dorsal auditory field containing a complete high-to-low tonotopic gradient running along the rostral-to-caudal axis. A tonotopic reversal at the anterior periphery of A1 was defined as a border between A1 and AAF. Tone-responsive areas that abutted a ventral border of A1 and posterior border of AAF were labeled as the VAF/SRAF. These fields had longer latency response than the A1 and AAF did. PAF and DAF were defined posteriorly and dorsally to A1, respectively, both with longer peak latency. AVAF was defined on the basis of tonotopic discontinuity at a ventral border of AAF and anteroventral border of SRAF.

To visualize the topography of the auditory cortex, the Voronoi tessellation procedure was used to create tessellated polygons with their centers corresponding at recording sites. Functional parameters such as CF and field were then illustrated by color-coded polygons. These polygons were used to characterize the area assigned to the functional parameters on the surface of auditory cortex.

The mutual information (MI) can be computed between a set of stimulus set, *S*, and a particular feature of neural responses, *R*, as follows:
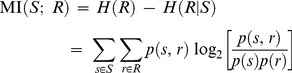
(1)where *p*(.) denotes a probability. *H*(*R*) and *H*(*R|S*) are the response entropy and noise entropy, respectively, each of which is defined by
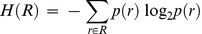
(2)

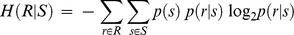
(3)


where *p*(*r|s*) denotes the probability of observed response *r* given presentation of stimulus *s*. The stimulus sets, *S*, consisted of 162 test stimuli with 2 stimulus parameters: 18 frequencies and 9 intensities. A frequency distribution of spike counts in a given temporal window of 10 ms was obtained for each test stimulus with a bin size of 1 ms. The temporal window was slid by 1-ms increments to examine the time course of MI, and the maximum value in this trace was taken as the representative MI.

Each neural response property of interest was characterized with respect to the functional map of the CF and field. Within a given representational area, the variances of MI, peak firing rate and latency were quantified by interquartile ranges (IQR). Additionally, after removing outliers, which were larger than the 75th percentiles or smaller than the 25th percentiles by 1.5 times IQR, the maximum and minimum values were taken within the area under the test. For discrete properties of threshold (5-dB increment) and bandwidth (1/3-octave increment), the variances were quantified by the Shannon index of diversity (ID):
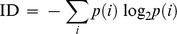
where *p*(*i*) is the proportion of individuals belonging to the *i*th discrete subgroup in the dataset; the threshold and bandwidth consisted of 7 subgroups (<30, 30, 35, 40, 45, 50 and >50 dB SPL) and 6 subgroups (1/3, 2/3, 1, 3/4, 3/5 and >2 octaves), respectively. ID is maximized when *p_i_* in each subgroup is identical.
